# Retraction Statement: Increased resting‐state activity in the cerebellum with mothers having less adaptive sensory processing and trait anxiety

**DOI:** 10.1002/hbm.26028

**Published:** 2022-07-23

**Retraction:** Sakakibara, N., Makita, K., Hiraoka, D., Kasaba, R., Kuboshita, R., Shimada, K., Fujisawa, T. X., & Tomoda, A. (2021). Increased resting‐state activity in the cerebellum with mothers having less adaptive sensory processing and trait anxiety. Human Brain Mapping, 42(15), 4985– 4995. https://doi.org/10.1002/hbm.25594. The above from Human Brain Mapping, published online on 16 July 2021 in Wiley Online Library (wileyonlinelibrary.com), has been retracted by agreement between the authors, the journal Editor‐in‐Chief, Simon B. Eickhoff and Wiley Periodicals, LLC. The retraction has been agreed given the journal has received evidence confirming that the peer review process of this paper was manipulated. As a result, the conclusions reported in the article are not considered reliable.

